# Influence of perinatal antibiotic on neonatal gut microbiota: a prospective cohort study

**DOI:** 10.1186/s12887-025-05907-y

**Published:** 2025-07-21

**Authors:** Faiza Iqbal, Padmaja A. Shenoy, Leslie Edward S. Lewis, N. Siva, Jayashree Purkayastha, Vandana Kalwaje Eshwara

**Affiliations:** 1 Department of Pediatrics, Kasturba Medical College, Manipal, Manipal Academy of Higher Education, Manipal, Udupi District, Manipal, Karnataka 576104 India; 2 Department of Microbiology, Kasturba Medical College, Manipal, Manipal Academy of Higher Education, Manipal, Udupi District, Manipal, Karnataka India; 3 Department of Child Health Nursing, SUM Nursing College, Siksha ‘O’ Anusandhan University, Bhubaneshwar, Odisha India

**Keywords:** Perinatal antibiotic exposure, Neonatal gut microbiota, Preterm neonates, Microbial diversity, Gut Microbiome

## Abstract

**Introduction:**

Given the significant role of the gut microbiota in early immune and metabolic development, the impact of perinatal antibiotic administration on the neonatal gut microbiome remains a crucial area of investigation. This study examines how maternal and neonatal antibiotic exposure affects the composition of the gut microbiome in preterm infants.

**Methodology:**

A prospective controlled cohort study was conducted in the neonatal intensive care unit of a tertiary care hospital from January 2021 to September 2023. The study enrolled neonates with a gestational age of less than 37 weeks. Preterm infants were categorized into four groups based on exposure to maternal or neonatal antibiotics: the NE group (no exposure), IE group (infant exposure only), ME group (maternal exposure only), and IME group (both infant and maternal exposure). In the NE group, “No exposure” refers to participants whose mothers did not receive intrapartum antibiotics or whose neonates were not administered postnatal antibiotics prior to sample collection. Data on antibiotic use and microbiota composition from stool samples were collected and analyzed via the convection culture method.

**Results:**

This study included 182 preterm infants, yielding 364 stool samples. By day 4, the prevalence of *Klebsiella pneumoniae* increased significantly, reaching 70% in the IE group compared with 42.6% in the NE group (*p* < 0.001). *Bifidobacterium spp*. was more prevalent in the NE group than in the other groups on day 4 (57.4%, *p* = 0.019). The antibiotic-exposed groups (IE, ME, and IME groups) presented greater abundances of potentially pathogenic bacteria, such as *Klebsiella pneumoniae* and *Escherichia coli*, whereas beneficial bacteria, such as *Bifidobacterium* spp., were more abundant in the NE group.

**Conclusion:**

These findings suggest that perinatal antibiotic exposure is associated with significant changes in the neonatal gut microbiota, potentially increasing the pathogenic microbiota.

**Trial registration:**

The study was registered with the Clinical Trials Registry-India (CTRI) under the number CTRI/2020/11/029375 |http://ctri.nic.in/ on November 19, 2020.

## Introduction

The overutilization of antibiotics has garnered significant attention in human medical research because of its associated adverse health implications and the emergence of antibiotic resistance [1, 2]. Concurrently, this concern extends to the perinatal period, where exposure to antibiotics plays a critical role in disturbing the neonatal gut microbiome, impacting various aspects of neonatal growth, immune system development, and susceptibility to diseases [3]. According to previous studies, approximately 80% of pharmaceutical prescriptions for pregnant women involve antibiotics, and recent figures indicate that between 20% and 40% of pregnant women worldwide receive these medications [4]. Antibiotic treatment profoundly affects the diversity and ecological composition of the gut microbiome in neonates, particularly in preterm infants. This disruption creates an imbalance in the microbial community, impairing gut barrier function and hindering the maturation of the immune system [5, 6].

Antibiotic administration to mothers during pregnancy or delivery can alter the maternal microbiota, which is a crucial source of microbial colonization affecting breastfeeding and vertical microbial transmission [7, 8]. These changes can extend beyond the vaginal and intestinal microbiomes, impacting the microbiomes of the mother’s milk and skin, which are vital for microbial transmission after delivery through breastfeeding and skin-to-skin contact [9, 10]. Intrapartum antibiotic prophylaxis, which is commonly used during cesarean sections and in mothers with group B Streptococcus colonization, reaches the fetus via the umbilical cord and alters the maternal vaginal and gut microbiome, influencing neonatal oral and gut microbial colonization [11, 12]. This disruption can lead to altered transmission of bacteria to neonates, initiating a chain reaction of microbiome imbalance in infants. Early perturbations in the gut microbiota, characterized by reduced microbial diversity and an increased prevalence of opportunistic pathogens in neonates, contribute to the weakening of intestinal defenses [13, 14]. This alteration in the gut ecosystem includes a decrease in beneficial bacteria such as *Lactobacillus* spp. and Bifidobacteriaceae and an increase in potentially harmful Proteobacteria [15]. These shifts can affect mucosal integrity and increase inflammation risk, underscoring the need for judicious use of antibiotics during perinatal care to balance infection prevention with maintaining healthy neonatal microbial development [4, 16].

When managing bacterial infections during pregnancy, the selection of appropriate antimicrobial therapy must balance efficacy with safety considerations for both the mother and the developing fetus [17]. Among the pharmacotherapeutic options commonly employed during pregnancy are penicillin’s (e.g., amoxicillin, ampicillin), cephalosporins (e.g., cephalexin), and macrolides (e.g., erythromycin, azithromycin) [18]. These antimicrobial agents are generally regarded as safe for maternal use during gestation and are frequently employed as first-line treatments for various bacterial infections encountered during pregnancy [19]. Importantly, however, prenatal antibiotic exposure can disrupt the maternal gut microbiota, potentially leading to alterations in the neonate’s gut microbiota following delivery [20]. This disruption may have implications for the establishment of the infant’s microbiome and subsequent health outcomes. Healthcare providers should exercise meticulous deliberation when prescribing antibiotics during pregnancy, balancing the potential benefits of treatment against any associated risks to maternal and fetal health, thereby ensuring optimal clinical outcomes [21].

The primary aim of this study was to investigate how exposure to antibiotics during the perinatal period influences the composition of the neonatal gut microbiota via culture-based methods. To generate robust evidence concerning the impact of perinatal antibiotics on the developing gut microbiota, we initiated a prospective controlled cohort study. This study scrutinizes the effects of maternal antibiotic prophylaxis, postnatal intravenous antibiotics, or a combination of both on the gut microbiome and the rise in antimicrobial resistance in preterm neonates admitted to the neonatal intensive care unit (NICU). Additionally, we gathered data on maternal antibiotic use during pregnancy, categorizing antibiotics on the basis of their mode of action.

## Methodology

### Study design and study population

A prospective controlled cohort study was carried out in which neonates were recruited from the NICU of a tertiary care hospital located in Karnataka, India. Preterm infants (gestational age < 37 weeks) with birth weights < 2500 g were enrolled after written informed consent was obtained from their parents between January 2021 and September 2023. Infants with congenital gastrointestinal anomalies diagnosed at birth, a confirmed diagnosis of necrotizing enterocolitis (NEC) prior to or within 48 h of sample collection, or blood culture-negative infections with clinical signs of sepsis were excluded. Additionally, to address concerns regarding the development of NEC after sample collection, neonates were monitored for NEC development for up to 28 days, and those diagnosed with NEC during this period were excluded from subsequent analyses. Infants whose samples were insufficient or degraded for microbiome analysis were also excluded.

### Ethical approval

This study was approved by the institutional ethics committee (IEC: 490/2020) and registered with CTRI (CTRI/2020/11/029375).

### Antibiotic exposure assessment

**Fig. 1:**
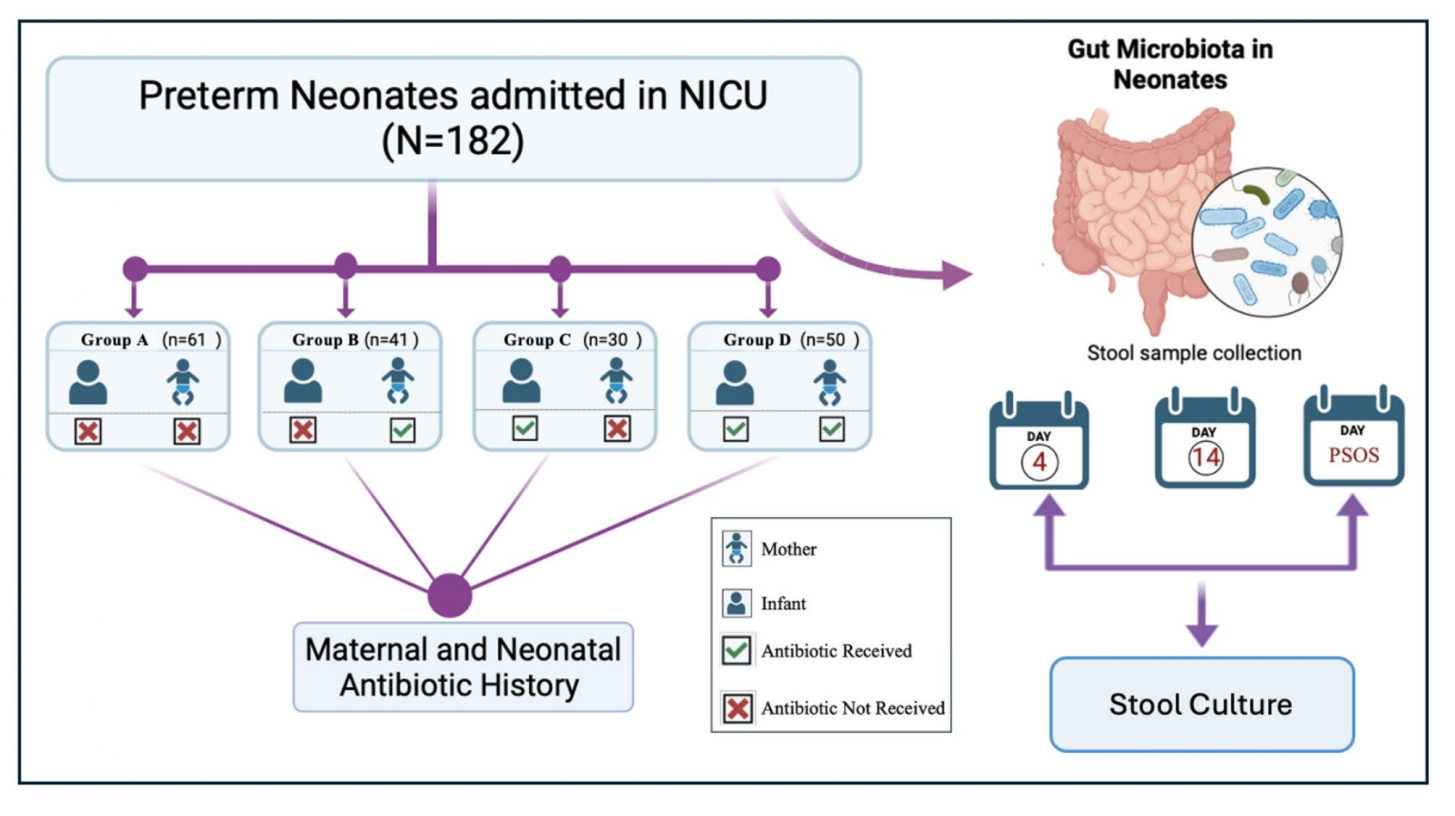
Outline of the study

The study categorizes participants into four distinct groups based on their exposure to antibiotics (Fig. 1): no exposure (NE), infant exposure (IE), maternal exposure (ME), and infant and maternal exposure (IME). The NE group comprises neonates with no exposure to maternal antibiotics within six months before delivery and no postnatal antibiotics in the first week of life. The IE group consisted of neonates who were administered postnatal intravenous antibiotics starting within the first 15 days after birth. The ME group comprises infants who experienced exposure to maternal antibiotics within six months before delivery. The IME group included neonates exposed to both maternal antibiotics and postnatal intravenous antibiotics. The antibiotics used for maternal and neonatal treatments were systematically classified according to their mechanism of action. In this study, the gut‒blood microbial correlation is defined as the concurrence of similar bacteria in both the gastrointestinal tract and the bloodstream.

### Data collection

All eligible inborn preterm infants admitted to the NICU were recruited at birth (day 1) and followed prospectively, observing them over time to identify the occurrence of sepsis. The neonates were followed prospectively for 15 days until the onset of sepsis. After the follow-up period, we divided the infants into groups based on sepsis development and the antenatal exposure of their mothers to antibiotics. Demographic details of the recruited neonates, such as sex, gestational age, birth weight, and morbidities, were collected from case records. Detailed maternal antibiotic exposures were collected from comprehensive case sheets recorded by the study physician. Information regarding blood culture and antimicrobial susceptibility patterns was retrieved from the hospital’s software Laboratory Information Services. For Group NE and Group ME, the first stool sample was taken on day 4 of life, and the second sample was taken on day 14. For Group IE and Group IME, the first stool sample was collected on the fourth day of life, whereas the second sample was obtained two days after the Gram stain of the blood culture bottles revealed the growth of pathogenic gram-negative bacteria, confirming sepsis. This time point is referred to as post sepsis onset sampling (PSOS). Stool samples were aseptically collected from the diapers of the neonates via a sterile spoon and transferred into sterile containers. These samples were transported within four hours to a microbiology laboratory for conventional culture workup, which included both aerobic and anaerobic cultures.

### Microbiome analysis

The samples from all the groups were transported to the microbiology laboratory within 4 h for optimal integrity. For qualitative aerobic culture, the samples were inoculated onto 5% sheep blood agar (Catalog No. MP1301, HiMedia Labs, Mumbai, India) and MacConkey agar plates (Catalog No. MPH081, HiMedia Labs, Mumbai, India), which are ideal for broad-spectrum gut bacterial growth. The plates were incubated at 37 °C, which is conducive to many bacterial species, and checked for growth at 24 and 48 h. The bacterial isolates were identified via matrix-assisted laser desorption/ionization time-of-flight mass spectrometry (MALDI-TOF MS). These isolates also underwent culture sensitivity testing via the VITEK 2 automated system. We determined antimicrobial susceptibility patterns via guidelines from the Clinical and Laboratory Standards Institute (CLSI, 2021), ensuring the application of current standards for comprehending resistance patterns and guiding treatment strategies. For anaerobic culture, the stool sample was divided into two parts. One part was inoculated onto 5% sheep blood agar and neomycin blood agar supplemented with a metronidazole disc (5 U). The plates were then incubated at 37 °C for 72 h in a Whitley A35 anaerobic workstation (Don Whitley Scientific, Shipley, UK). The second part of the stool sample was inoculated into Robertson’s cooked meat (RCM) medium and incubated at 37 °C. Gram staining was performed on the 3rd, 5th, and 7th days of incubation, and if any new bacterial morphology was observed, subcultures were performed on 5% sheep blood agar. The bacterial isolates obtained from these cultures were identified via MALDI-TOF MS.

### Statistical analysis

Patient demographics are summarized as the means ± standard deviations (SDs) for normally distributed data and as medians with interquartile ranges (IQRs) for skewed distributions. Categorical variables such as bacterial prevalence and antibiotic susceptibility are expressed as percentages. We used the chi-square test to assess the associations between maternal and neonatal antibiotic administration and gut-blood microbial correlation across the four groups. Antibiotic resistance data were descriptively analyzed, stratified by group and timepoint (day 4 vs. PSOS), and presented via frequency tables and proportions to show shifts in resistant organisms over time. Statistical analyses were conducted via the SPSS Version 16.0 package (IBM Corp. IBM SPSS Statistics for Windows, Armonk, NY).

## Results

A total of 364 stool samples were collected from 182 preterm infants. Among these, 61 neonates in the NE group (mothers not, infants not) were not exposed to perinatal antibiotics. Additionally, 41 infants were classified into Group IE (infants exposed, mothers not), 30 into Group ME (mothers exposed, infants not), and 50 into Group IME (both mothers and infants exposed). The baseline characteristics of the neonates and mothers included in the study are presented in Table 1. Empirical broad-spectrum antibiotics were initiated upon clinical suspicion of sepsis and collection of blood cultures. Definitive antibiotic therapy was adjusted based on the results of antimicrobial susceptibility testing (AST), typically available after 24–48 h. The day definitive antibiotic therapy started is defined as the antibiotic exposure day, with a mean age of exposure of 8 ± 6.15 days.

**Table 1:** Baseline characteristics of the neonates and mothers included in the study (*N* = 182)

Neonatal characteristics	Group NE (*n* = 61)	Group ME (*n* = 41)	Group IE (*n* = 30)	Group IME (*n* = 50)
Birthweight(grams)^a^	1436.4 ± 372.7	1722.3 ± 653.2	1448.6 ± 434.4	1786.9 ± 658.1
Gender, *n* (%)				
Female	26(42)	11(26.8)	15(50)	19 (38)
Male	35(57.3)	30(73.1)	15(50)	31(62)
Maternal parity *n* (%)				
Primigravida	31(50.8)	21(48.7)	17(56.6)	23(46)
Multigravida	30(49)	21(51.2)	13(43.3)	27(54)
Mean gestational age^a^	31.3 ± 2.19	30.6 ± 3.37	31.3 ± 1.74	32 ± 4.6
Gestational age, *n* (%)				
Extremely preterm (< 28 Weeks)	8(13.1)	6(14.6)	1(3)	2(4)
Very preterm (28 to 32 Weeks)	35(57)	23(37.7)	19(63.3)	26(46)
Late preterm (33 to 37 Weeks)	19(31)	12(29.2)	10(33.3)	23(46)
Neonatal growth outcome, *n* (%)				
LGA	1(1.6)	0(0)	0(0)	1(2)
AGA	46(75.4)	33(80.4)	21(70)	37(74)
SGA	14(22.9)	8(19.5)	9(30)	12(24)
Mode of delivery, *n *(%)				
Vaginal delivery	9(14.3)	14(31.4)	7(23)	11(22)
Cesarean delivery	52(85.3)	27(65.8)	23(76)	39(78)
Mode of feeding, *n* (%)				
Breastfeeding	20 (32.7)	12(29.2)	10(33.3)	17(34)
Formula feeding	23(37.7)	14(34.1)	10(33.3)	21 (42)
Mixed feeding	18 (29.5)	15 (36.5)	10(33.3)	12 (24)
Maternal characteristic				
Mean age of mothers^a^	30.1 ± 4.2	30.2 ± 4.4	31.6 ± 3.7	31.9 ± 4.6
Infections treated with antibiotics *n* (%)				
Urinary tract infection	0	0	14 (46)	13 (26)
Vaginitis	0	0	4 (13)	11 (22)
Pneumonia	0	0	1 (3)	1 (2)

*LGA* large for gestational age, *AGA* appropriate for gestational age, *SGA* small for gestational age

^a^Values represented as the mean ± standard deviation

### Microorganisms isolated from blood cultures and antimicrobial susceptibility profiles of bacterial isolates

In neonates diagnosed with sepsis (*n* = 91), analysis of bacterial isolates from blood cultures revealed that 81.3% (74) of the pathogens were resistant to multiple drugs, whereas 18.7% (17) were sensitive to the antibiotics tested. *Klebsiella pneumoniae* was the most frequently isolated bacterium, accounting for 39.5% (36) of the total isolates, highlighting its dominant role in neonatal infections in this study population. The next most prevalent organisms were *Acinetobacter baumannii* and *Escherichia coli*, accounting for 19.8% (18) and 13.2% (12) of the isolates, respectively, indicating their substantial presence in neonatal sepsis patients. Other notable pathogens included *Staphylococcus spp*. and *Acinetobacter nosocomialis*, each constituting 8.7% (08) and 3.3% (03) of the isolates, respectively. Fewer common bacteria, such as *Enterococcus faecium*, *Enterococcus faecalis*, and various species of *Pseudomonas* spp., *Streptococcus* spp., *Enterobacter* spp., and *Achromobacter* spp., were also identified, each contributing less than 3.3% to the total bacterial profile.

**Fig. 2:**
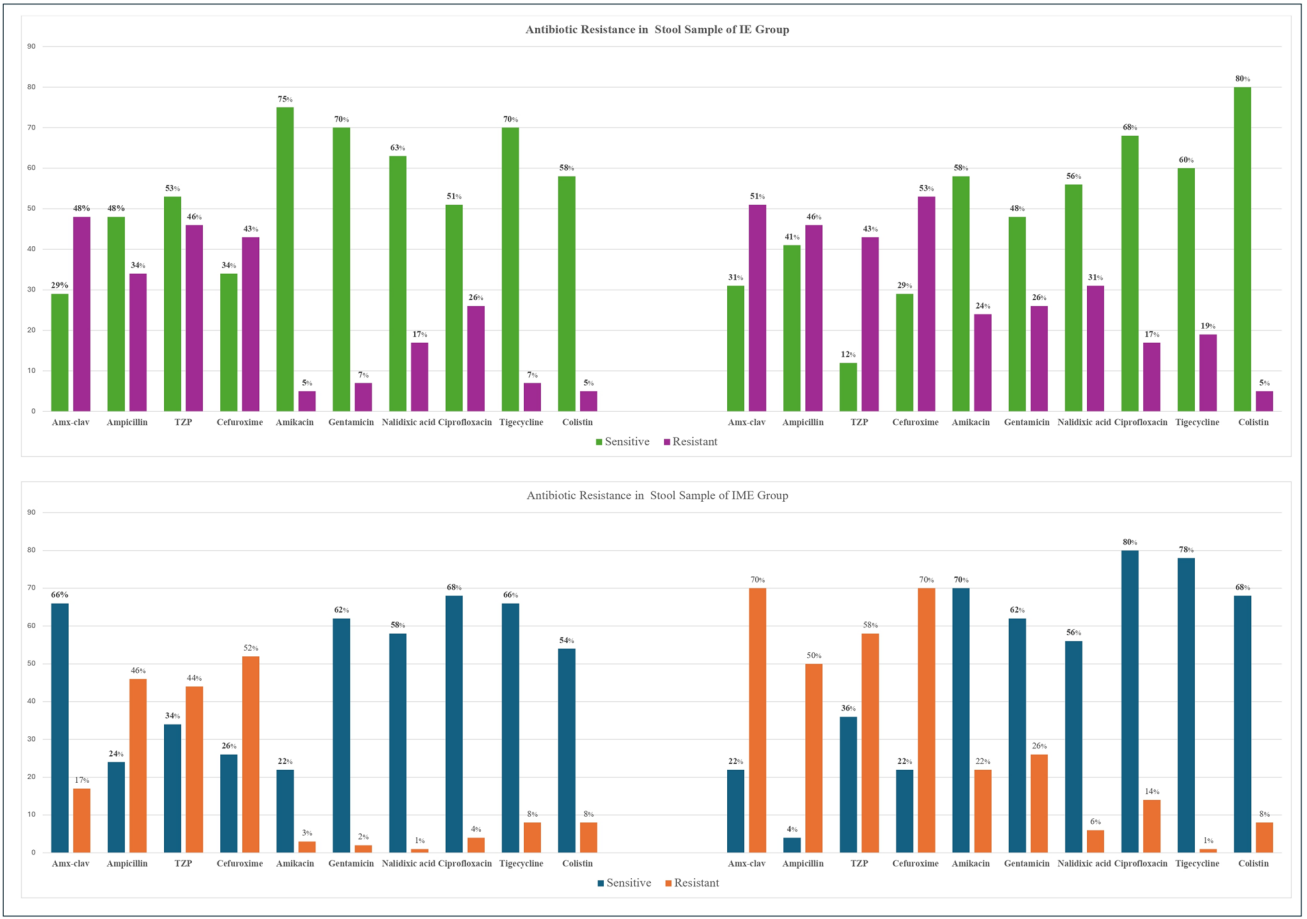
Antibiotic resistance in stool samples of the IE and IME groups

Antimicrobial susceptibility testing of bacterial isolates from neonatal blood cultures revealed various resistance profiles across different antibiotics. Among the tested antibiotics, gentamicin had the highest sensitivity, with 47.3% of the isolates susceptible to gentamicin, followed by trimethoprim/sulfamethoxazole and amikacin, with sensitivities of 45.1% and 42.9%, respectively. On the other hand, ampicillin and cefuroxime had the highest resistance rates, with 48% of the isolates resistant to both antibiotics. Similarly, cefotaxime/ceftriaxone and cefepime/cefepime also presented high resistance rates, with 45% and 39% of the isolates resistant, respectively. Intermediate resistance was generally low across all antibiotics, with ciprofloxacin/ofloxacin having the highest intermediate resistance rate at 4.4%. Similar intermediate resistance rates were observed for cefoperazone-sulbactam and cefepime, each at 4.4%. Meropenem and imipenem, representing the carbapenem class, had substantial sensitivity rates of 37.4% and 30.8%, respectively but also had notable resistance rates of 33% and 34.1%, respectively. Antibiotic resistance data from the stool samples from the IE and IME groups are shown in Fig. 2.

### Maternal antibiotic use

In Group ME, we observed a varied pattern of antibiotic usage among the participating mothers. The use rates of amoxicillin, clindamycin and piperacillin/tazobactam were 13.3% each, which is indicative of their roles in managing bacterial infections that are of particular concern during pregnancy. Moderate usage of nitrofurantoin (12%) and metronidazole (3.3%) was documented, aligning with their prescribed roles in the urinary tract and certain bacterial infections, respectively. Less frequently used antibiotics include tinidazole (6.6%), ceftriaxone (6.6%), and azithromycin (3.3%), which are generally reserved for specific indications. In Group IME, urinary tract infections contributed to 26%, whereas vaginitis was present in a smaller proportion, affecting 22% of the mothers. The antibiotics Co-trimoxazole, clindamycin, and piperacillin/tazobactam were the most administered antibiotics, each of which was used in 13.3% of the instances, reflecting their relevance in managing the observed infections. The occurrence of maternal complications varied without dominance of any condition, indicating that a broad range of health issues were encountered within the cohort.

### Influence of maternal antibiotic administration on neonatal gut microbiota composition

**Fig. 3:**
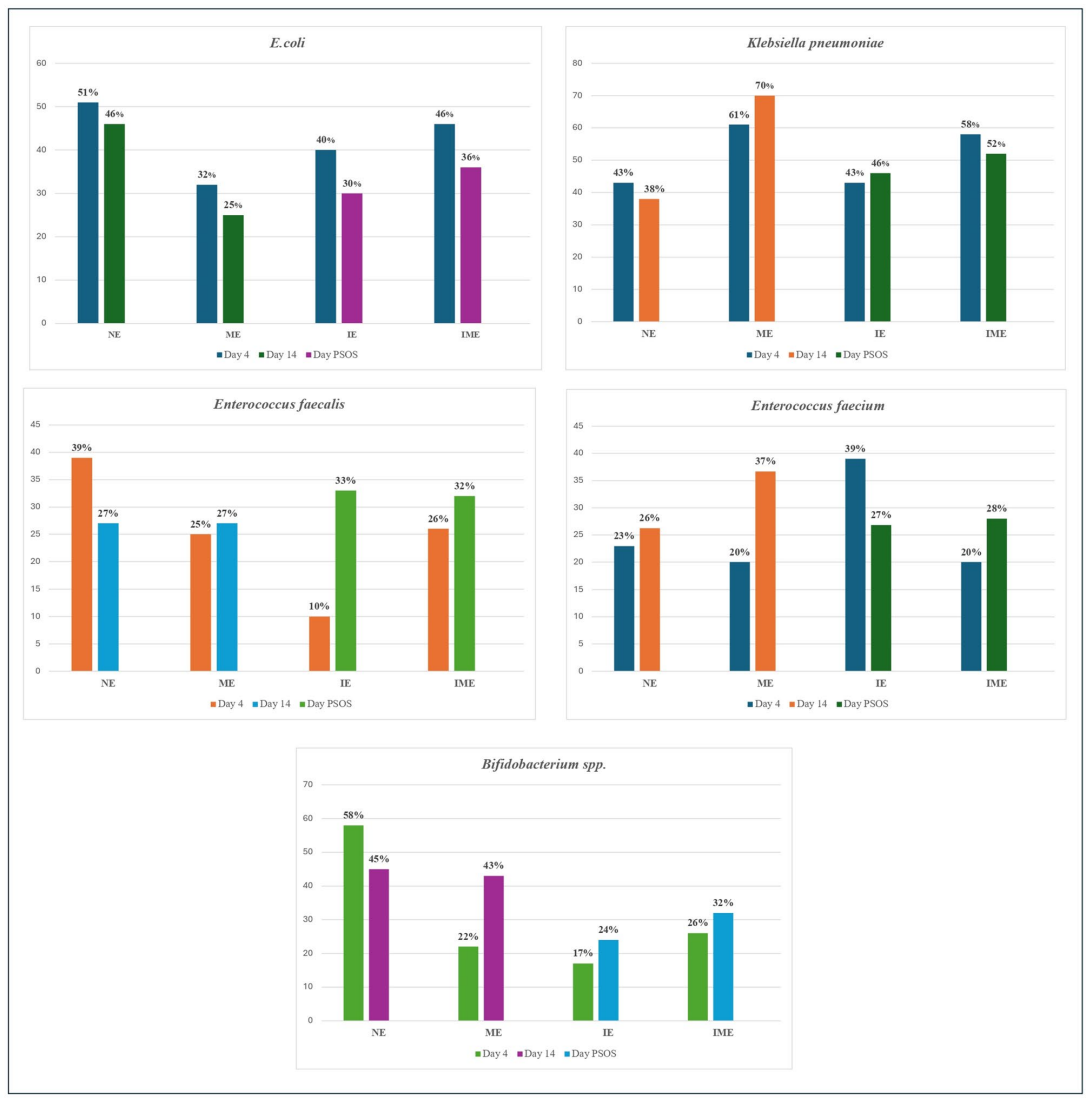
Comparison of the gut microbiota composition on day 4 and day 14 of life across different groups

In Group ME, the prevalence of specific gut microbiota among infants at days 4 and 14 of life is depicted in Fig. 3. Compared with the control group (Group NE), the ME group correlated with altered neonatal gut composition, even in the absence of direct neonatal antibiotic treatment. The infants in group IE, who directly received antibiotics, presented the most significant alterations, suggesting a strong impact of direct antibiotic exposure on the neonatal gut microbiota. The IME group, with combined maternal and neonatal antibiotic exposure, exhibited an even broader range of microbial shifts, highlighting the compounded effects of antibiotics. These findings emphasize the importance of antibiotic stewardship in perinatal care to preserve healthy neonatal gut microbiota, which is crucial for the development of the immune system and metabolism.

The chi-square test was utilized to determine the associations between the groups receiving maternal antibiotics and those not receiving maternal antibiotics. On Day 4, the prevalence *of Klebsiella pneumoniae* was 52.5% (42) in neonates whose mothers received antibiotics, whereas it was 47.5% (38) in the control group. This difference was not statistically significant (*p* = 0.261). A statistically significant difference was observed for *Klebsiella pneumoniae* on Day 14, where it was found in 63.7% (51) of the antibiotic group (Group ME) compared with 36% (25) of the non-antibiotic group (Group NE) (*p* < 0.001). For *Bifidobacterium* spp. on day 4, a significant association was found, where 41% (33) of the antibiotic group had this bacterium, whereas 58% (47) of the non-antibiotic group did (*p* = 0.019). However, the difference in the prevalence of *Bifidobacterium spp*. on day 14 was not significant (48.9% (24) with antibiotics vs. 51.1% (56) without antibiotics, *p* = 0.67).

### Neonatal antibiotic use

In this cohort, which included two groups (Group IE and Group IME), a total of 91 neonates received antibiotic treatment. We categorized the antibiotics based on their mode of action, specifically those that affect cell wall synthesis, cell membrane integrity, protein synthesis, and nucleic acid synthesis. Overall, there was a heavy reliance on beta-lactams (particularly penicillin’s) and aminoglycosides (predominantly amikacin) for managing neonatal infections. Non-beta-lactams are less commonly used but remain an important component of the neonatal antibiotic arsenal. Table 2 presents the classification of antibiotics based on their mode of action. Among neonates who received colistin, *Klebsiella* spp. was the most prevalent bacterial species, detected in 59.5% of cases on Day 4 and increasing to 75.7% on PSOS days. *E. coli* was present in 80% of the cases on Day 4, whereas *E. faecium* and *E. faecalis* were detected in 27.0% of the PSOS cases. Similarly, in neonates administered amikacin, *Klebsiella* spp. remained the dominant microbe, with a prevalence of 58.1% on day 4 and 74% on the day of PSOS. *E. coli* was detected in 38.7% of the cases on Day 4, whereas *E. faecium* and *E. faecalis* were detected in 29.0% and 25.8% of the cases, respectively, by PSOS.

**Table 2:** Antibiotic usage in neonates (according to mode of action) (*N* = 91)

Inhibit		Classification	Antibiotics	*n* (%)
Cell wall synthesis	Beta Lactams	Penicillin	Piperacillin/Tazobactam	74 (81.3%)
			Ampicillin	69 (75.8%)
		Cephalosporins	Ceftriaxone	3 (3.2%)
			Cefoperazone	4 (4.3%)
			Cefixime	1 (1.09%)
			Cefotaxime	2 (2.19%)
		Carbapenems	Meropenem	8 (8.7%)
	Non Beta Lactams	Glycopeptide Antibiotics	Vancomycin	4 (4.3%)
Cell membrane	Colistin			9 (9.8%)
Protein synthesis	30 S	Aminoglycosides	Amikacin	85 (93.4%)
			Gentamycin	3 (3.2%)
	50 S	Macrolides	Erythromycin	1 (1.09%)
Nucleic acid synthesis	DNA Topoisomerases	Fluoroquinolones	Ciprofloxacin	6 (6.5%)

### Correlations between gut-blood microbial correlations and antibiotic exposure

The causative organism constituted more than 10% of the gut microbiota. Notably, in 75% of these cases, stool samples presented over 45% bloodstream infection-causing species. The most identified organisms in both blood and stool samples included *Klebsiella pneumoniae*,* Escherichia coli*, and *Enterobacter* spp. This significant presence of the causative bacteria in the gut prior to infection suggests an association between gut colonization and subsequent bloodstream infection. Group IE exhibited a gut‒blood microbial correlation in 10 (24%) samples on day 4, which increased slightly to (11) 26% on the PSOS day. In comparison, Group IME, which consisted of neonates exposed to both maternal antibiotic prophylaxis and postnatal antibiotics, demonstrated higher rates of gut–blood microbial correlation, with 20 (40%) samples on day 4 and 18 (36%) on PSOS day exhibiting this characteristic. These findings suggest a potential impact of antibiotic exposure, both prenatally and postnatally, on the composition and integrity of the neonatal gut microbiome, possibly influencing the translocation of bacteria from the gastrointestinal tract to the bloodstream.

### Influence of mode of feeding on neonatal gut microbiota composition in antibiotic-exposed neonates (Groups IE and IME)

In the IME group, distinct differences in gut microbiota composition were observed on Days 4 and PSOS day based on the mode of feeding. On Day 4, *Bifidobacterium spp*. showed the highest relative abundance in breastfed neonates (76%), followed by mixed-fed (60%) and formula-fed infants (45%). *Enterococcus faecalis* was also notably abundant across all feeding types, particularly in breastfed (63%) and formula-fed infants (56%). Interestingly, *E. coli* showed its highest prevalence in mixed-fed neonates (59%) compared to formula-fed (33%) and breastfed infants (45%). By PSOS day, *Bifidobacterium spp.* further increased in breastfed infants (80%) and remained high in mixed-fed (65%) but showed a relative decline in formula-fed neonates (45%). *Enterococcus faecalis* continued to be dominant, especially in mixed-fed (69%) and breastfed (66%) groups. Notably, *E. coli* prevalence declined across all groups, most markedly in mixed-fed infants (26%). These findings suggest that exclusive breastfeeding in antibiotic-exposed dyads supports the colonization of beneficial bacteria such as *Bifidobacterium spp.* and *Enterococcus faecalis*, while formula feeding is associated with higher relative abundance of Enterococcus faecium and reduced bifidobacterial colonization.

In Group IE, mode of feeding significantly influenced gut microbiota colonization patterns on both Day 4 and PSOS day. On Day 4, *Bifidobacterium spp.* showed the highest abundance in breastfed infants (58%), followed by mixed-fed (52%) and formula-fed (47%) neonates. *E. coli* was notably abundant in mixed-fed neonates (61%). *Enterococcus faecalis* levels were highest in breastfed (45%) and mixed-fed (45%) groups, whereas *Enterococcus faecium* was more pronounced in formula-fed infants (38%). By PSOS day, a marked increase in *Bifidobacterium spp.* was observed in breastfed infants (80%) and mixed-fed neonates (55%), whereas a decrease was seen in formula-fed infants (36%). *E. coli* abundance rose further in mixed-fed infants (72%) and remained relatively high in breastfed (63%) and formula-fed (54%) groups. *Enterococcus faecalis* and *Enterococcus faecium* remained consistently represented across all feeding types, with slightly elevated levels in formula-fed and mixed-fed infants.

**Fig. 4:**
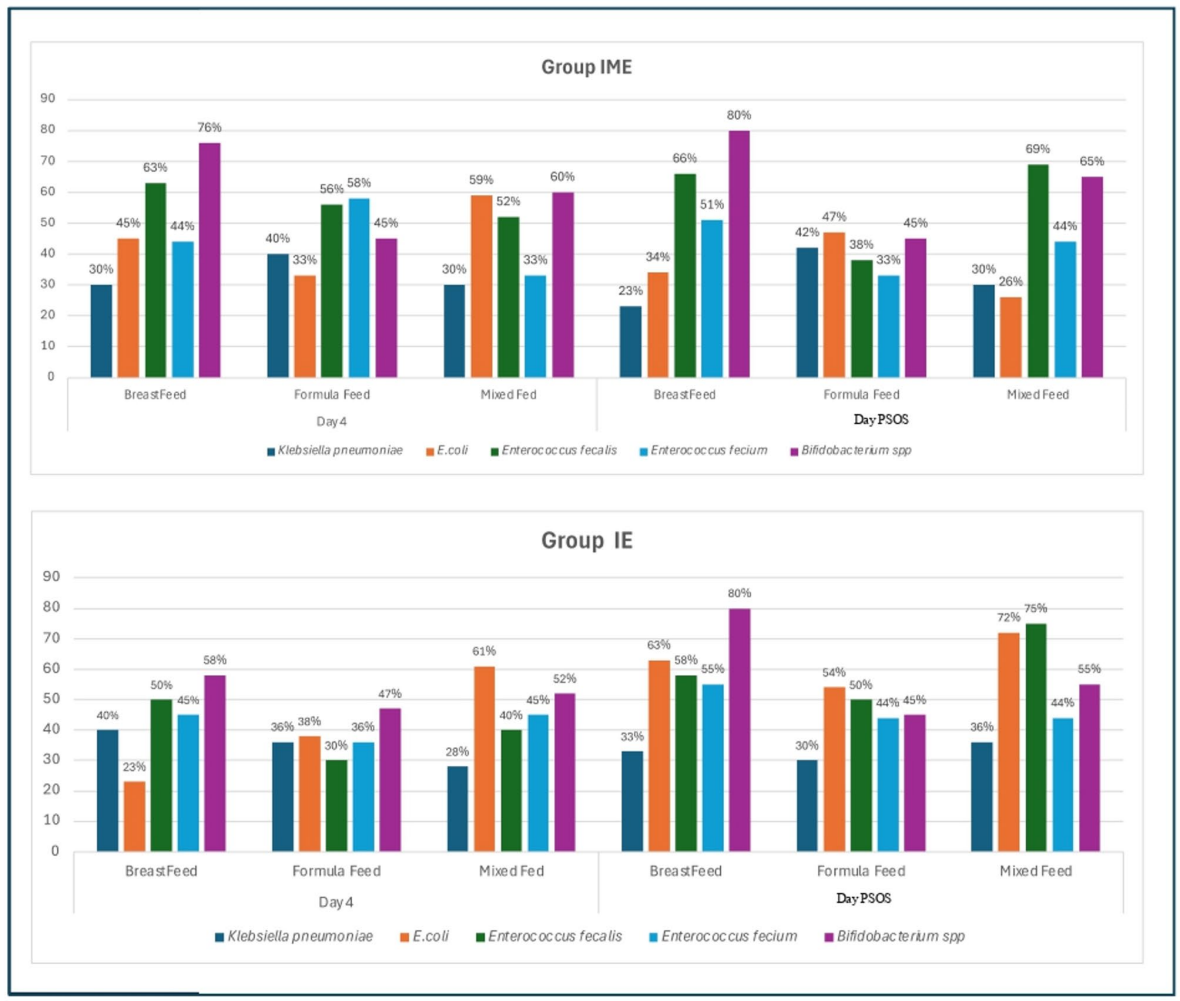
Influence of feeding mode on gut microbiota composition in antibiotic-exposed neonates (IME vs. IE groups)

When comparing the two groups, Group IE (infant-only antibiotic exposure) showed higher relative abundance of *E. coli* by PSOS day, specifically in mixed-fed infants (72%) compared to Group IME (26%). Additionally, Group IE exhibited better colonization with *Bifidobacterium spp.* in breastfed infants (80%)—a trend that was also present in Group IME (80%) but with lower levels in formula-fed neonates. *Enterococcus faecalis* was consistently abundant across both groups, suggesting its resilience post-antibiotic exposure. Overall, combined maternal and neonatal antibiotic exposure (Group IME) appeared to result in greater dysbiosis, with decrease in *E. coli* diversity and delayed Bifidobacterium recovery in non-breastfed infants. This highlights the protective role of exclusive breastfeeding, particularly in maintaining beneficial gut flora following antibiotic perturbation (Fig. 4).

## Discussion

This study contributes to the burgeoning field of perinatal antibiotic exposure and its subsequent effects on the neonatal gut microbiome. In the present study, *Bifidobacterium* spp. levels varied across groups, indicating the impact of antibiotic exposure. In the NE group, there was an increased proportion of *Bifidobacterium spp*. on day 14, suggesting a healthier gut microbiota and the suppression of potentially pathogenic bacteria such as *Klebsiella pneumoniae* and *Escherichia coli*. However, antibiotic exposure reversed this trend, resulting in reduced levels of *Bifidobacterium* spp. and increased abundance of potentially pathogenic bacteria in both the perinatal and postnatal exposure groups. The direct and combined effects of antibiotics were evident, as both the IE and IME groups presented a lower proportion of *Bifidobacterium* spp. over time, whereas *Klebsiella* spp. and other potentially harmful bacteria became more dominant. Notably, by day 14, the NE and ME groups presented similar levels of *Bifidobacterium* spp., highlighting a possible recovery trajectory in the absence of continued antibiotic pressure. This increase suggests that maternal antibiotic exposure can indirectly influence the neonatal gut microbiome, even without direct antibiotic administration to infants. These findings are in line with those of Morreale et al. (2023), who discussed the broader implications of perinatal antibiotic exposure on neonatal gut health and composition [1]. A study by Uzan-Yulzari et al. (2021) also revealed that neonatal antibiotic exposure can impair child growth during the first six years of life, which is associated with significant changes in the gut microbiome, especially a decreased abundance of *Bifidobacterium* spp [22]. Gasparrini et al. (2019) emphasized that early-life antibiotic treatment and hospitalization lead to long-lasting changes in the microbiome and resistance in preterm infants. In preterm infants, persistent strains of *E. coli* (*n* = 139), *Klebsiella pneumoniae* (*n* = 62), *Enterococcus faecalis* (*n* = 50), *Enterobacter cloacae* (*n* = 42), *Enterococcus faecium* (*n* = 22), *Citrobacter. freundii* (*n* = 15), and *Klebsiella oxytoca* (*n* = 14) were detected, in stool culture both during the NICU stay and after discharge [23]. Similarly, in this study, *Klebsiella pneumoniae*, *Escherichia coli*, and *Enterococcus faecium* were the predominant bacteria isolated from the stool cultures of preterm neonates in the IME group on the day of PSOS, indicating a shift toward a more pathogenic microbiota composition following antibiotic exposure.

In the present study, urinary tract infections (UTIs) were the predominant reason for maternal antibiotic prescription, accounting for 33.7% (27) of the cases, which aligns with the findings of a global survey, where UTIs were the most frequently treated infection at 67.9% [4]. The findings of this study offer an insightful comparison of the maternal antibiotic usage patterns reported for the ME and IME groups, shedding light on regional and cohort-specific variations in antibiotic prescribing practices during pregnancy. Stokholm et al. (2022) noted the predominant use of macrolides (2%), nitrofurantoin (1%), penicillin (15%), and ampicillin derivatives (6%) [24]. This study revealed a comparable trend with specific antibiotics such as cotrimoxazole, clindamycin, and piperacillin/tazobactam, each of which was administered to 13.3% of the participants in both the ME group and the IME group. This consistent use underscores the critical role these antibiotics play in managing infections during pregnancy, reflecting targeted approaches to antibiotic therapy on the basis of their effectiveness and safety profiles [25].

The antibiotic usage patterns observed in this study align closely with those reported by Chandra et al. (2023), where amikacin and piperacillin-tazobactam were the most frequently prescribed definitive therapies. Specifically, piperacillin-tazobactam was administered in a significant majority of cases (81.3%), mirroring findings from this study in both the IE group and the ME group [26]. Additionally, the high usage rate of ampicillin (75.8%) corroborates the emphasis on the use of penicillin in managing infections. In contrast, cephalosporins such as ceftriaxone, cefoperazone, cefixime, and cefotaxime showed markedly lower utilization rates, suggesting a more selective approach for their application. The limited use of non-beta-lactams, such as vancomycin and colistin, highlights their role in treating more resistant infections. This pattern underlines the critical need for tailored antibiotic stewardship programs to manage resistance and optimize therapeutic outcomes. These findings suggest that antibiotic intervention can lead to a reduction in beneficial gut bacteria, highlighting the importance of considering the effects of antibiotic use on the microbiota of both mothers and their offspring. This research contributes to the growing body of evidence on the impact of antibiotics on neonatal health and development, reinforcing the need for stringent guidelines and judicious use of antibiotics in perinatal care to safeguard against unintended consequences on neonatal gut microbiota and overall child health.

## Potential implications for neonatal health and the rationale behind the observed microbial shifts

Early-life antibiotic use can lead to significant and profound shifts in the gut microbiota composition of neonates. These gut alterations can disrupt and cause dysbiosis in the natural colonization process, which is critical for the development of the immune system and metabolic functions. Altered microbial composition and the suppression of beneficial bacterial populations such as *Bifidobacterium* spp. can impair gut barrier function, increasing susceptibility to infections and inflammatory conditions such as neonatal sepsis and necrotizing enterocolitis. The rationale behind these observed microbial shifts stems from the broad-spectrum nature of antibiotics, which, while targeting pathogenic bacteria, also indiscriminately affect commensal microorganisms. These findings underscore the importance of antibiotic stewardship and of further research into the potential benefits of approaches to correct dysbiosis.

## Limitations

We acknowledge the limitations inherent in culture-based methods for analyzing microbial diversity and function, which are relevant to understanding the neonatal gut microbiota. These methods inherently select for microorganisms that can be cultivated under laboratory conditions, representing only a small fraction of the microbial community. As a result, the evaluation of the complete diversity and functional potential of the gut microbiota remains limited, potentially omitting key microbial populations and their ecological roles. This limitation underscores the importance of integrating culture-independent approaches, such as metagenomics and high-throughput sequencing, to achieve a more comprehensive assessment of microbial composition and function. These advanced methodologies allow for the characterization of uncultivable microbial taxa and provide critical insights into the dynamic interactions within the gut microbiome that may be influenced by perinatal antibiotic exposure. Future studies utilizing such approaches may provide deeper insights into the nuanced effects of antibiotics on the neonatal gut microbiota.

## Conclusion

This study offers significant insights into the effects of perinatal antibiotic exposure on the development of the neonatal gut microbiota. This study highlights the importance of antibiotic stewardship, which involves careful consideration of antibiotic use during pregnancy and early neonatal life to balance the benefits of infection management against potential adverse effects on the gut microbiome.

## Data Availability

Data is provided within the manuscript and additional data is available on request to the corresponding author.
